# Supplementary material for: Computational analysis of tongue reconstruction surgery: The impact of donor site and flap volume on post-operative anatomy and biomechanics
Source: PLoS One. 2026 Mar 26;21(3):e0345755. doi: 10.1371/journal.pone.0345755 (PMC13021171; doi:10.1371/journal.pone.0345755)
Supplement: Attachment — Submitted filename: Response-Letter-PONE-D-25-59556.pdf [file pone.0345755.s003.pdf]

From:
Amir R. Isazadeh
PhD in Rehabilitation Sciences, Communication Sciences & Disorders
Faculty of Rehabilitation Medicine,
University of Alberta.
Email: isazadeh@ualberta.ca
Feb 20, 2026

To:
Alice Berardo
Academic Editor
PLOS One

Re: Response to Revisions for Manuscript PONE-D-25-59556

Dear Dr. Berardo,
Thank you for the opportunity to revise our manuscript, "Computational analysis of tongue reconstruction surgery: the impact of free flap site and volume on the tongue anatomy and biomechanics" (Manuscript ID: PONE-D-25-59556). We are grateful to you and the reviewers for the constructive feedback.

All points raised by editors and reviewers have been carefully addressed. The manuscript has been improved as a result, and a revised version with tracked changes (highlights) has been submitted, along with a clean version and the updated source files.

A point-by-point response to each of the original comments is provided below for clarity, and revised sentences and paragraphs are highlighted. It is our hope that these revisions and responses have satisfactorily addressed all concerns. The manuscript is now stronger, and we hope it will be considered suitable for publication in Surgical Oncology.

Thank you for your time and consideration.
Sincerely,
Amir R. Isazadeh
(Corresponding Author)
On behalf of all co-authors.

Journal Requirements:

1. Please ensure that your manuscript meets PLOS ONE's style requirements, including those for file naming. The PLOS ONE style templates can be found at https://journals.plos.org/plosone/s/file?id=wjVg/PLOSOne_formatting_sample_main_body.pdf and https://journals.plos.org/plosone/s/file?id=ba62/PLOSOne_formatting_sample_title_authors_affiliations.pdf

Response:

Thank you for the provided links. We have updated the manuscript to strictly comply with the journal's formatting guidelines.

- All major headings (Level 1) have been adjusted to 18pt bold, and subsections (Level 2) to 16pt bold, ensuring a clear visual hierarchy.
- Every heading has been converted to sentence case and unnumbered.
- Updated all captions to ensure titles are in bold, followed by a non-bolded descriptive legend.
- Relocated figure and table captions to appear immediately following the paragraph where they are first cited.
- Standardized all in-text mentions to use the "Fig" abbreviation and corrected figure file naming to align with journal requirements.
- Formatted all figure and table captions with bold titles followed by standard-type legends.
- Relocated captions to appear immediately after the paragraph of their first citation.
- Updated all in-text references to use compliant "Fig" and "Table" notation, including Supporting Information (e.g., "S1 Fig").
- Verified and corrected the chronological order of all figure and table mentions.
- Optimized cross-referencing for Supporting Information sections.
- All display and inline equations have been standardized (full compliance with the journal’s mathematical formatting requirements).
- Inline mathematical expressions were revised to ensure that punctuation and non-mathematical text are positioned outside the math environment.
- Statistical reporting, including p-values and model parameters, has been uniformly formatted for consistency.
- Formatted Supporting Information Citations as "S1 Fig", etc. Cited multiple files as "S1 and S2 Figs", "S1-S3 Figs", etc.
- Table legends and footnotes are corrected to be compliant.
- Numbers and symbols of the affiliation converted to superscript.
- Listed corresponding author’s initials in parentheses after the email address.

2. We note that you have indicated that there are restrictions to data sharing for this study. For studies involving human research participant data or other sensitive data, we encourage authors to share de-identified or anonymized data. However, when data cannot be publicly shared for ethical reasons, we allow authors to make their data sets available upon request. For information on unacceptable data access restrictions, please see http://journals.plos.org/plosone/s/data-availability#loc-unacceptable-data-access-restrictions.

Before we proceed with your manuscript, please address the following prompts:

a) If there are ethical or legal restrictions on sharing a de-identified data set, please explain them in detail (e.g., data contain potentially identifying or sensitive patient information, data are owned by a third-party organization, etc.) and who has imposed them (e.g., a Research Ethics Committee or Institutional Review Board, etc.). Please also provide contact information for a data access committee, ethics committee, or other institutional body to which data requests may be sent.
b) If there are no restrictions, please upload the minimal anonymized data set necessary to replicate your study findings to a stable, public repository and provide us with the relevant URLs, DOIs, or accession numbers. Please see http://www.bmj.com/content/340/bmj.c181.long for guidelines on how to de-identify and prepare clinical data for publication. For a list of recommended repositories, please see https://journals.plos.org/plosone/s/recommended-repositories. You also have the option of uploading the data as Supporting Information files, but we would recommend depositing data directly to a data repository if possible.
Please update your Data Availability statement in the submission form accordingly.

Response:
We are committed to open science and have made the complete simulation framework, code, and all necessary input files (including the segmented geometries) publicly available to ensure full reproducibility of our results. These resources are hosted on GitHub (https://github.com/is-Amir/VSP-BioSim) and archived with a citable DOI (10.5281/zenodo.17438091). However, the "raw" MRI data cannot be publicly shared. The specific ethical and legal restrictions are detailed below:

The restrictions are imposed by the "Health Research Ethics Board of Alberta (HREBA)" under Protocol HREBA.CC-17-0597. Because the dataset consists of high-resolution head and neck MRI scans, it inherently contains facial soft tissue data. Even if standard de-identification (removal of metadata/DICOM tags) is performed, the 3D volumetric data can be surface-rendered to reconstruct the participant's face, making the data potentially identifiable.

Consequently, public deposition of this raw data would violate the terms of the participant's informed consent and patient privacy standards.

This restriction aligns with standard practices in the field and recent publications within the PLOS ecosystem. For example, similar restrictions were accepted for “The G-OBIM tongue model: An accurate open-source biomechanical model of a male human tongue” (PLOS Computational Biology, 2025, doi: 10.1371/journal.pcbi.1013378).

Researchers may request access to the de-identified MRI data. Requests may be directed to the corresponding author or the ethics committee at the address below:

Health Research Ethics Board of Alberta – Cancer Committee
1500, 10104 - 103 Avenue NW
Edmonton, Alberta, T5J 0H8, Canada
Telephone: +1 (780) 423-5727
Fax: +1 (780) 429-3509
Email: cancer@hreba.ca

Revised “Data and code availability” section:
The simulation framework is made openly available [54] under MIT license, without restriction, enabling full reproducibility of all 120 simulations and direct application to new patient cases. The ”Raw” MRI images cannot be publicly shared due to privacy restrictions imposed by the Health Research Ethics Board of Alberta. However, all inputs required to reproduce the simulation outcomes (including the segmented geometries) and statistical results are included in this repository (hosted on GitHub at https://github.com/is-Amir/VSP-BioSim and archived with a citable DOI: 10.5281/zenodo.17438091). Access to de-identified MRI data may be granted to researchers upon request to the corresponding author or the Health Research Ethics Board of Alberta – Cancer Committee (cancer@hreba.ca).

3. If the reviewer comments include a recommendation to cite specific previously published works, please review and evaluate these publications to determine whether they are relevant and should be cited. There is no requirement to cite these works unless the editor has indicated otherwise.

Response:
Thank you for clearing this matter.

Reviewer #1:

The authors present a comprehensive work that provides a deep evaluation, through numerical simulations, of different scenarios and aspects related to tongue reconstruction surgery, with a particular focus on the donor site and flap volume. The topic is relevant, but clarifying certain methodological aspects and adding further discussion would enhance the manuscript before publication.

Response:
We thank the reviewer for their positive assessment and appreciate the constructive feedback. We believe the manuscript has been significantly strengthened by incorporating these suggestions.

Comment:
1 INTRODUCTION
- The authors state that tongue mobility after reconstruction is the most important aspect to preserve, and they describe and compare the characteristics of two donor sites: the Radial Forearm Free Flap and the Anterolateral Thigh. To provide better context for the study, it would be useful to provide a brief overview of the donor site choices commonly used in the literature, along with their pros and cons. This would highlight the advantages of the Radial Forearm and Anterolateral Thigh Free Flaps chosen for investigation and help readers understand their relevance to surgical outcomes.

Response:
Providing a broader overview of available donor sites is indeed essential to contextualize our specific focus on the Radial Forearm and Anterolateral Thigh flaps. We have added a concise paragraph acknowledging alternative sites. This addition sets the stage for the study's biomechanical analysis.

Added paragraph:
"Preserving residual tongue mobility is the foremost determinant of functional recovery; mobility for speech and swallowing precedes bulk. Although alternative donor sites, such as the rectus abdominis (RA) and latissimus dorsi (LD), are used for massive defects, the Radial Forearm Free Flap (RFFF) and Anterolateral Thigh (ALT) flap are predominant in practice, representing the biomechanical spectrum from thin, pliable tissue to substantial bulk [1, 2, 8, 9]. "

Comment:
2 METHOD
2.1 Patient-Specific Tongue Model
- The tongue model was derived from an MRI scan of a healthy male participant in his 40s.

Including participant characteristics, such as height and/or BMI, would help better understand the model's tongue dimensions and those of the free flap under consideration.

Response:
Thank you for this suggestion. To protect the participant's identity, we have maintained the generalized age descriptor ("in his 40s") rather than including specific anthropometric data such as exact height and weight. However, we have added the participant's BMI (30.4 kg/m2) to Section "Patient-specific tongue model" to provide the requested context regarding the model's dimensions.

Comment:
- You state that: "To ensure consistency across representative cases, we adopted the same four clinically plausible tongue reconstruction scenarios and used "resection models" identical to those in our prior work". Please provide the reference for the work you are referring to and briefly describe the four scenarios for clarity.

Response:
Thank you for this important observation. The specific nature of the resection scenarios was not sufficiently defined in the original text. We have revised this section to explicitly cite the prior work and structurally describe the four cases in Fig1.

Revised paragraph:
"To span a representative spectrum of Urken class I–II tongue defects [2], four resections were computationally simulated as detailed previously [7] and described in (Fig 1)."

"Fig 1. Patient-specific tongue model, resection scenarios, and flap designs. Overview of the four simulated tongue cancer cases: (A) tip-sparing hemiglossectomy; (B) lateral hemiglossectomy; (C) posteriorly extended hemiglossectomy sparing the tongue base; and (D) asymmetric, midline-crossing partial glossectomy."

Comment:
- The patient-specific tongue model comprises the tongue, the resected tongue and the jaw. Please specify which software and procedures were used to extract the tongue model from MRI, obtain the resected tongue (through Boolean subtraction) and construct the rigid jaw models.

Response:
We appreciate the reviewer's request for greater technical specificity regarding the model generation workflow. Listing the specific software tools is essential for reproducibility. We have revised this section to explicitly state that Mimics Medical 17.0 was used for the initial MRI

segmentation of the tongue and mandible, while Blender was utilized for the Boolean resection operations and the elastic registration of the jaw model.

Revised sentences:
“This study is built upon a high-resolution, patient-specific 3D model of the tongue, previously segmented using Mimics Medical 17.0 (Materialise, Leuven, Belgium) from an MRI scan of a healthy male participant (BMI: 30.4 kg/m2) in his 40s [7].”

“For each case, the “resected tongue model” was generated via a Boolean subtraction of the corresponding resection volume from the baseline tongue anatomy using Blender (Blender Foundation, Amsterdam, Netherlands).”

“The mandibular surface was first segmented from the patient’s MRI data using Mimics Medical. Subsequently, a high-resolution generic jaw template was registered to this surface using an elastic mesh deformation algorithm in Blender, guided by manually placed anatomical landmarks.”

Comment:
- Figure 1: It would improve the readability to indicate the letter of the four different panels (A-B) in the figure captions. In addition, please define the hiFEM acronym in the figure caption or within the text, explain why it is used, and describe how the planar flap design was obtained.

Response:
Thanks for the opportunities to improve the clarity of Figure 1. We have revised the figure and caption as follows:

1- Panel Labels: We have added explicit labels (A–D) to the figure panels and updated the caption to clearly define each resection scenario: tip-sparing hemiglossectomy (Case A), lateral hemiglossectomy (Case B), posteriorly extended hemiglossectomy (Case C), and asymmetric midline-crossing partial glossectomy (Case D).

2- hiFEM Definition: We have ensured the acronym hiFEM (Hyperelastic Inverse Finite Element Method) is defined in the text within the "Biomechanically optimized flap design" section.

3- Methodology Description: We clarified in the text that hiFEM is an established algorithmic tool developed in our previous work, used here to deterministically generate the planar flap designs. To maintain a focused narrative, we treat the algorithm as a verified input generator for this study, referring readers to the cited methodology for the mathematical derivation, while briefly noting its function (generating optimized planar shapes from 3D defects) in the caption and in the specified section.

Revised sections:

"Fig 1. Patient-specific tongue model, resection scenarios, and flap designs. Overview of the four simulated tongue cancer cases: (A) tip-sparing hemiglossectomy; (B) lateral hemiglossectomy; (C) posteriorly extended hemiglossectomy sparing the tongue base; and (D) asymmetric, midline-crossing partial glossectomy. The biomechanically optimized planar flap designs are generated using the hiFEM algorithm, as detailed in previous work [7]. The texture maps each point in the resection model to its corresponding point in the planar flap design."

"The process begins by employing the hiFEM (hyperelastic inverse Finite Element Method), as detailed in previous work [7], to produce a biomechanically optimized planar skin template for each resection case"

Comment:

2.2 Biomechanically Optimized Flap Design

- Figure 2A is not cited in the main text; please include an explicit reference to it.

Response:

We have revised the opening sentence of the "Biomechanically optimized flap design" section to explicitly reference Fig 2A, identifying the resection model as the geometric basis for the flap generation process.

Revised sentence:

"As an input for the surgical simulation, a patient-specific three-dimensional free flap model is generated from the resection model (Fig 2A), which is composed of skin and adipose layers (Fig 2B–D)."

Comment:

- Given the procedural workflow, in my opinion, it may improve the clarity to invert the order of panels B and C in Figure 2.

Response:

We appreciate the reviewer's suggestion to align the figure panels with the procedural workflow, as the generation of the 2D template (Panel C) indeed conceptually precedes the 3D realization (Panel B). However, we respectfully propose retaining the current layout, as the panels were intentionally arranged to function as an orthographic projection to assist the reader in visualizing the complex 3D geometry of the flap. In this arrangement:

- Panel B provides a side view of the volumetric layers.
- Panel C provides the frontal/planar view (the 2D template).

- Panel D provides the top view (showing thickness and overbulking).

We believe prioritizing this spatial arrangement allows the reader to better grasp the volumetric structure of the flap design. To address the reviewer's concern regarding clarity, we have updated the figure caption to explicitly identify these viewing angles, ensuring the reader understands the relationship between the panels.

Revised caption:
"Fig 2. … optimized 2D planar skin template for the flap (C: flap's frontal view). This template's deterministic 3D-to-2D mapping then guides the computational "unfolding" of the resection model to generate a multi-component free flap model (B: flap's side view). To simulate the clinical practice of "overbulking," the flap's volume is increased by scaling the thickness of its yellow adipose component (D: flap's top view) … "

Comment:
- Figure 2: Panels F and G represent the anterior and posterior views of the model; this should be specified in the figure caption to improve clarity.

Response:
Thanks for the suggestion. We have updated the figure caption to explicitly identify Panel F as the anterior view and Panel G as the posterior view.

Revised caption:
"Fig 2. … tongue's muscular attachments to the mandible and hyoid bone, shown in anterior (F) and posterior (G) views."

Comment:
2.3 Simulation of Tongue Reconstruction Surgery
- Please explicitly state in this section the software (FEBio) adopted to perform the computational simulation of the surgical procedure for tongue reconstruction.

Response:
We have updated the opening sentence of Section "Simulation of tongue reconstruction surgery" to explicitly state that the surgical simulation was performed using the open-source finite element solver FEBio:
"The surgical procedure of tongue reconstruction was computationally simulated using the open-source finite element solver FEBio [40]"

Comment:
- The author states that "Following the virtual suturing, a subsequent step allows the system to

equilibrate for five minutes". Please clarify the rationale for selecting this time interval. Was the choice based on previous studies, experimental tests, or literature evidence?

An insightful question regarding the temporal definitions in our simulation protocol; Thank you. The selection of the 300-seconds equilibration interval was intentional. It was determined quantitatively based on the specific viscoelastic time constants of the tissues modeled in this study, derived from the experimental literature cited in Table 1. To ensure the "Day 0" state represented a stable, post-surgical equilibrium, we analyzed the decay of the shear relaxation modulus, $G(t)$, for each tissue type using their respective Prony series parameters. We calculated the **Percent Relaxation Complete** at $t = 300s$, defined as the proportion of the total possible transient stress decay that has occurred: $(G(0) - G(300)) / (G(0) - G_{\infty})$. The results confirm that a 5-minute interval is sufficient for the system to reach mechanical stability:

- **Subcutaneous Adipose Tissue:** The relaxation process is effectively **100%** complete (longest time constant $\tau = 10s$).
- **Skin:** The tissue has undergone **97.8%** of its total stress relaxation.
- **Native Tongue:** The tissue has undergone **97.1%** of its total stress relaxation.

This analysis demonstrates that extending the simulation beyond five minutes would yield negligible changes in the stress state ($< 3\%$), as the system has effectively reached its quasi-static equilibrium. Thus, 300 seconds provide a robust, computationally efficient, and scientifically justified duration to establish the baseline for long-term remodeling.

We have updated the manuscript (Section: *Simulation of tongue reconstruction surgery*) to explicitly state this quantitative justification:

“Following the virtual suturing, a subsequent step allows the system to equilibrate for five minutes. This duration was quantitatively chosen by evaluating the decay of the shear relaxation modulus, G(t), using the viscoelastic parameters detailed in Table 1. The analysis confirmed that >95% of the total transient stress relaxation had completed within this interval (97.1% for tongue, 97.8% for skin, and 100% for adipose tissue).”

Comment:
2.6 Constitutive Models
- In the manuscript, it is reported that “All soft tissues (tongue, skin, and subcutaneous adipose tissue) were modelled as nearly incompressible, non-linear hyperelastic materials.” In my opinion, given the description of the tissue properties in the paragraph (the choice of Yeoh and Ogden models for the hyperelastic component and the introduction of viscous behaviour), it would be more accurate to state that “All soft tissues (...) were modelled as nearly incompressible, isotropic, non-linear hyperelastic and time-dependent materials”.

Response:
Thank you for this precise suggestion. We have revised the sentence in Section "Constitutive Models" to: "All soft tissues (tongue, skin, and subcutaneous adipose tissue) were modeled as nearly incompressible, isotropic, nonlinear, hyperelastic, and time-dependent materials."

Comment:
- Considering the parameters reported in Table 1, the constitutive models adopted to describe the behaviour of the tissues are a 2-term Yeoh model for the native tongue, a 2-term Ogden model for the skin, and a 1-term Ogden model for the adipose tissue. Since not all models include 2 terms, it may be clearer in equations (1) and (2) to report the summation over i from 1 to N and then specify the values of N for each tissue.

Response:
Thank you for catching this. We have revised Equations (1) and (2) to imply the summation limit N, which corresponds to the number of terms. To provide further clarity, we have updated Table 1 to explicitly list the value of N for each tissue's constitutive model (e.g., $N = 2$ for native tongue and skin hyperelasticity, and $N = 1$ for adipose tissue). This approach keeps the mathematical formulation general while providing the specific implementation details in the summary of material parameters.

Added sentence:
"... N denotes the number of terms in the summation. The specific values of N used for each tissue type are detailed in Table 1."

Comment:
2.7 Soft Tissue Parametrization
- The authors report that, to account for the high experimental variability in tissue stiffness for both Forearm and Thigh skin, a boundary approach was adopted, resulting in three representative uniaxial stress-stretch curves for each donor site. However, the rationale for this approach and the criteria used to select the three reported curves are not clearly described. Please provide additional details to clarify this choice.

Response:
We agree that the criteria for selecting these constitutive models needed to be explicitly defined to justify the experimental design. Our rationale stems from the significant inter-subject variability observed in literature reports, where stiffness curves can vary substantially. Therefore, we defined the solution space of possible surgical outcomes. We have revised Section "*Soft tissue parameterization"* and Figure 4 to explicitly define our selection criteria:

- For thigh skin: We utilized the comprehensive dataset from Zhou et al. (2024). The three curves are defined statistically: the mid-stiffness level corresponds to the experimental mean, while the high- and low-stiffness levels correspond to the mean + 1 Standard Deviation (STD) and mean - 1 STD, respectively.
- For Forearm Skin: The low-stiffness parameters were calibrated to the most compliant data reported (e.g., Flynn et al., 2011), while the high-stiffness parameters were calibrated to the stiffest responses reported (e.g., Griffin et al., 2017 and Jacquet et al. 2017), with an intermediate curve acting as the central tendency.

This approach enables our factorial study to cover the entire spectrum of clinically realistic tissue behaviors.

Revised Text for Section *Soft tissue parameterization*:

"To investigate the impact of donor-site selection, material parameters for forearm and thigh skin were derived from a thorough literature review (Fig 4). Given the substantial stiffness variability observed within and between donor sites, we defined three distinct stiffness levels (low, mid, and high) for each site to encompass the physiological range. For thigh skin, parameters were derived from the statistical data reported by Zhou et al. [43]. The mid-stiffness model was calibrated to the study's mean stress–stretch curve, while the high- and low-stiffness models were calibrated to the mean ± standard deviation (SD) to rigorously bound biological variance. For forearm skin, where experimental observations vary significantly between studies, we captured the average and extremes of reported stiffness. Low-stiffness parameters were calibrated to the most compliant experimental data [44] and high-stiffness parameters to the stiffest reported responses [45, 46], with the mid-stiffness level representing the central tendency of the aggregated literature."

Revised Figure 4 Caption:

"Fig 4. Literature review of reported donor skin material properties. (i) forearm skin: The solid lines illustrate the three calibrated stiffness levels—low (green), medium (blue), and high (red)—spanning the range of experimental data reported in the literature (dashed lines [44–47]). (ii) thigh skin: Stiffness levels were derived from Zhou et al. [43]. The experimental mean (mid-stiffness, blue), mean + SD (high-stiffness, red), and mean - SD (low-stiffness, green) were used to capture the physiological range of tissue variability (shaded region)"

Comment:

- Figure 4: The stiffness levels adopted for both the forearm and thigh skin are three; however, while all three curves are shown for the forearm, only two curves are reported for the thigh. Please also report the three representative uniaxial stress-stretch curves for the thigh site. In addition, the three stiffness-level curves for the forearm are difficult to distinguish; consider using, e.g., different colours or line styles. Finally, the x-axis is labelled as "Steretch"; please correct it to "stretch".

Response:
We have updated Figure 4 to address these visual improvements:

- Thigh skin (panel ii): All three stiffness levels are now explicitly plotted. As detailed in the response above, these curves represent the experimental mean and the mean ± STD bounds.
- Forearm visibility (panel i): We assigned unique colors (green, blue, red) and increased line thickness to clearly distinguish the three simulation levels from the background literature data.
- Labeling: The x-axis label has been corrected to "Stretch". Thank you!

Comment:
- The tissue properties were incorporated into the simulations, adopting a 2-term Ogden model calibrated to the three different stiffness levels (both for the forearm and thigh). Please describe in more detail how this calibration was performed, specifying which method/optimisation procedure was adopted.

Response:
Thank you for highlighting this. We have updated the manuscript to clarify that the material parameters were identified using a genetic algorithm optimization procedure implemented in Python (using the SciPy library). This global optimization approach was selected to robustly minimize the root-mean-square error (RMSE) between the analytical response of the Ogden model and the defined stress-stretch curves, ensuring the best fit for each of the six stiffness profiles.

Revised sentence: “... The calibration was performed using a genetic algorithm optimization procedure implemented in Python using the SciPy library. …”

Comment:
- Depending on the tissue, the number of viscous branches in the Prony series varies (4 for the native tongue and skin, 3 for the subcutaneous adipose tissue). Please specify the reason for this choice. Was it based on the literature or on the fitting/calibration procedure?

Response:
The number of viscous branches in the Prony series (N=4 for tongue and skin; N=3 for subcutaneous adipose) was adopted directly from the experimental characterizations reported in the literature, without modification. We utilized the specific viscoelastic parameters established by Vaidya et al. (2020) for the native tongue, Then et al. (2017) for the skin, and Sun et al. (2021) for subcutaneous adipose tissue. These values were selected to ensure the simulation's time-dependent behavior is grounded in validated experimental data without modifications/recalibration.

Comment:
- Table 1: for the subcutaneous adipose tissue, the parameter m1 should be dimensionless. Please verify and correct it.

Response:
Thank you for catching this typo. We have verified the parameter in Table 1, and you are correct: m1 for the subcutaneous adipose tissue (Ogden model) is a dimensionless exponent and should not have units of pressure. We have also investigated the other material parameters in the table to ensure no similar errors exist.

Comment:
2.8 Simulation Stability and Robustness
- The results of the mesh convergence analysis are reported in the Supplementary Material. However, the number of nodes and elements used in the models (e.g., average values or a range) should also be reported in the section for completeness.

Response:
Thank you for this suggestion. To ensure full clarity and transparency regarding the model discretization, we analyzed the mesh statistics across all 120 simulations included in the study. We have updated the text to explicitly report the mean, standard deviation, and range for both the number of nodes and elements observed in the final models.

Added sentences to the "Supporting information" section.
"Across all 120 simulations, the final models consisted of 25,532 ± 521 nodes (average ± standard deviation), ranging from 24,594 to 26,693. The corresponding number of elements was 20,858 ± 418 (range: 20,027–21,958)."

Comment:
2.9 Outcomes Metrics
- The authors referred to panels v and vi of Figure 3. To improve clarity, it may be better to split this figure into two separate figures: Figure 3 with panels i-iv and Figure 5 with panels v and vi. In addition, it should be specified in the Figure, near each contour, which metric is under consideration: effective strain or Hausdorff distance.

Response:
We agree with the reviewer that splitting the figure improves the logical flow and clarity of the manuscript. We have removed panels (v) and (vi) from Fig 3, which now focuses exclusively on illustrating the computational pipeline (steps i–iv).

The outcome metrics are now presented in a new, dedicated figure (Fig 5) at the beginning of the Results section. As requested, we have ensured that the specific metrics (Effective Strain and Hausdorff Distance) are clearly labeled and distinguishable in this new figure.

Comment:
3-4 RESULTS AND DISCUSSION
- Given that computational simulations are a crucial part of the study, it would be appropriate to include a discussion—and at least one representative figure—showing the main contours of effective strain and Hausdorff distance. While reporting all 120 simulations is not feasible, selected representative cases (e.g., specific reconstruction scenarios, tissue properties, and levels of overbulking) would be highly informative and enrich the manuscript.

Response:
We utilized the new Fig 5 to address this suggestion directly. This figure now displays the spatial distribution of the primary outcomes. To provide a concrete example of the simulation output, we selected a representative reconstruction scenario: Defect Case C reconstructed with a mid-stiffness forearm flap and 66.7% overbulking. This addition allows readers to qualitatively appreciate the results before delving into the statistical aggregation of the full 120-simulation dataset.

New Fig 5:
"Fig 5. Representative simulation results showing the spatial distribution of the two primary outcome metrics. (A) effective strain and (B) surface deviation (Hausdorff distance). These contours capture the end state of the neotongue for a representative reconstruction scenario: Defect Case C reconstructed with a mid-stiffness forearm flap and 66.7% overbulking."

Comment:
- The study considers three stiffness levels at the two donor sites: the forearm and the thigh. Although stiffness is included as a factor in the regression analyses, the manuscript does not explicitly report how changes in stiffness alone affect the output metrics (effective strain and Hausdorff distance). Discussing the effects of stiffness on these metrics would strengthen the paper.

Response:
We agree that isolating the effect of stiffness is critical. In our manuscript, we have quantified and discussed how increasing stiffness levels (within each donor site) impacts both determining metrics.

Regarding Effective Strain, the results of the main-effects regression model (Table 4) demonstrate that stiffness is a highly significant, independent predictor of strain. In the Results section, we report:
"All main effects—overbulking, is thigh, and stiffness level —were highly significant positive predictors of strain. Increasing flap volume, using tissue from the thigh, and increasing skin stiffness each independently and consistently increased the maximum effective strain experienced by the native tongue tissue."

We further interpret this in the Discussion section as the "tethering" effect:
“Our regression analysis (Table 3) reveals a clear, quantitative relationship: the stiffer the flap tissue, the greater the biomechanical strain imposed on the residual native tongue. … This biomechanical penalty was further amplified by increasing stiffness levels within each donor site.”

Regarding Hausdorff Distance (anatomical deviation), our interaction model (Table 3) revealed that while stiffness alone was not a significant main effect ($p = 0.125$), it played a crucial role in modulating the success of overbulking. We explain this in the Results section:
"…the beneficial effect of overbulking is significantly modulated by... stiffness level (coefficient = 0.160, $p < 0.001$)."

And discuss the physical implication in the Discussion section:
“This effect is reinforced by the overbulking × stiffness level interaction (coefficient = 0.160, $p < 0.001$), which reveals that for any given donor site, the anatomical return on investment from overbulking is greatest when using the most pliable tissue available (the lower stiffness level).”

We hope these clarifications highlight that the independent and interactive effects of stiffness have been analyzed and are integral to our conclusions.

Comment:
- Part of the study relies on a specific constitutive formulation; it would be useful for the Discussion to more explicitly comment on how the results might qualitatively change—or remain robust—if alternative constitutive models were adopted.

Response:
We agree that the choice of the hyperelastic strain energy function (specifically the Yeoh model for the tongue and the Ogden model for the flap tissues) is a critical modeling decision that warrants further discussion.

We have expanded the Discussion section to explicitly address the robustness of our results in the context of alternative constitutive models (e.g., Mooney-Rivlin or Neo-Hookean). Our argument for the qualitative robustness of the reported trends relies on three critical points:

First, all physically plausible hyperelastic models used in soft tissue biomechanics (including Yeoh, Ogden, and Mooney-Rivlin) share the same fundamental mathematical properties: they are monotonic and convex strain energy functions designed to capture the material's resistance to deformation. While the specific trajectory of the stress-strain curve differs between formulations, they all describe a material that stiffens with strain. Therefore, the relative ranking of our results (for instance, that a stiffer thigh flap induces greater tethering than a pliable forearm flap) is driven by this shared fundamental behavior and is unlikely to be reversed by the choice of a specific convex potential.

Second, we selected the Yeoh and Ogden formulations because they are widely recognized as the most effective phenomenological models for capturing the strong non-linear, strain-stiffening behavior characteristic of biological soft tissues. As noted in the biomechanical literature, the Yeoh model is particularly advantageous because it captures the typical J-shaped stress-strain response of lingual tissue more accurately than simpler models like the Neo-Hookean or Mooney-Rivlin, which often struggle to represent stiffening at large strains without instability. Furthermore, since the Neo-Hookean and Mooney-Rivlin formulations can be mathematically viewed as special cases or restricted derivations of the generalized Ogden behavior, our use of the more flexible Ogden and Yeoh forms allows for a superior fit to the experimental data used in our study.

Third, the choice of the Yeoh model is grounded in the specific history of tongue biomechanics. As reviewed by Hermant et al. (2017) the field has largely converged on the Yeoh model for finite element implementations (Gerard et al., 2005; Buchaillard et al., 2009; Stavness et al., 2012). This convergence is due to the Yeoh model's demonstrated ability to robustly fit experimental data from both ex vivo indentation and in vivo aspiration studies of the human tongue.

Finally, we argue that the primary trade-off observed in this study (between volume restoration and tissue strain) is mechanistically driven by tissue incompressibility. Regardless of the strain energy function, the conservation of volume dictates that overbulking a confined defect must result in geometric displacement and strain. Thus, while alternative models would undoubtedly alter the quantitative magnitude of the stress values, the qualitative mechanism and the identified clinical trade-offs are expected to remain robust.

Added paragraphs:

"The qualitative robustness of these findings extends to the specific choice of constitutive formulation. While this study utilized the Yeoh and Ogden models (selected for their efficacy in capturing the nonlinear stiffening of oral tissues [28]), alternative hyperelastic potentials would likely yield similar trends due to shared fundamental physics. Specifically, the reported trade-offs are mechanically driven by quasi-incompressibility and monotonic strain-stiffening; conservation of volume dictates that overbulking a confined space must invariably impose strain, while the universal convexity of valid soft tissue models ensures that the relative ranking of flap stiffness effects remains consistent regardless of the specific strain energy function employed."

References in this response:

Buchaillard, S., Perrier, P. and Payan, Y. (2009) "A biomechanical model of cardinal vowel production: Muscle activations and the impact of gravity on tongue positioning," *The Journal of the Acoustical Society of America*, 126(4), pp. 2033–2051.

Gerard, J.M., Ohayon, J., Luboz, V., Perrier, P. and Payan, Y. (2005) "Non linear elastic properties of the lingual and facial tissues assessed by indentation technique. Application to the biomechanics of speech production," Medical Engineering & Physics, 27(10), pp. 884–892.

Hermant, N., Perrier, P. and Payan, Y. (2017) "Human Tongue Biomechanical Modeling," in Y. Payan and J. Ohayon (eds.) *Biomechanics of Living Organs*. Oxford: Academic Press (Translational Epigenetics), pp. 395–411.

Stavness, I., Lloyd, J., Fels, S. (2012) "Automatic prediction of tongue muscle activations using a finite element model." J. Biomech. 45, 2841–2848.

Reviewer #2:

This study presents a robust, automated, and open-source computational testbed for evaluating interdependent surgical variables in free flap tongue reconstruction. By quantifying anatomy–biomechanics trade-offs through large-scale simulation, the framework provides physics-based insights into the consequences of donor site selection, flap stiffness, and overbulking. Its scalability and automation offer a pathway toward personalized, simulation-informed surgical planning, with the potential to improve functional outcomes in tongue reconstruction.

Response:
We thank the reviewer for their positive assessment. We appreciate their recognition of the framework's robustness and its potential to advance personalized, simulation-informed surgical planning through physics-based insights.

Before the Editor makes a decision, I suggest that the authors must take into account the following corrections:
1. I think the title needs to be reformulated to become more "friendly".

Response:
We appreciate the comment on improving the context of the title to make it more reader-friendly. To this end, we revised it with better wordings.

New title: Computational analysis of tongue reconstruction surgery: The impact of donor site and flap volume on post-operative anatomy and biomechanics

2. The present form of the abstract is a bit week not much clear. Hence, I recommend to re-write it with 3/4 stronger sentences about your objectives/ findings that will give a better understanding for the readers

Response:
We appreciate the reviewer's feedback regarding the clarity of the abstract's objectives and findings. To address this, we have refactored the abstract to explicitly state the study's goal. We also refined the results section to more clearly highlight the key findings. These changes significantly improve the readability and impact of the abstract.

Abstract:
Tongue reconstruction requires a series of decisions tailored to patient needs to restore anatomy and preserve speech and swallowing. The impact of these interdependent choices is difficult to evaluate, as clinical outcomes depend on case-specific factors. However, computational analysis offers a method for analyzing these interdependencies in a controlled fashion. The present study systematically quantifies the impact of key surgical decisions, namely donor site selection (radial forearm vs. anterolateral thigh) and flap volume on the final anatomical and biomechanical outcomes. To achieve this, we developed an automated framework that simulates free flap tongue reconstruction. The framework leverages biomechanically optimized flap design to generate a multi-component virtual flap, which is then computationally sutured to the resection site, and its long-term tissue atrophy is simulated to predict the final neotongue state. Using this platform, we conducted a 120-simulation factorial study for the systematic analysis. Four clinically plausible tongue reconstruction scenarios, six levels of flap stiffness, and five levels of flap overbulking (intentional excess volume) were simulated. The results reveal a fundamental, physics-based trade-off: while increasing flap overbulking was the dominant factor in restoring pre-operative anatomy, it came at the cost of a predictable increase in biomechanical strain imposed on the native tongue. Furthermore, stiffer flaps induced significantly higher baseline strain. The anatomical benefit of overbulking was significantly modulated by tissue properties, favoring

forearm flaps over thigh flaps. These findings provide a biomechanical rationale for clinically observed functional trade-offs. This work presents an open-source, robust computational testbed for systematically evaluating interdependent surgical variables. It provides quantitative, physics-based insights into the clinical trade-offs of surgical planning. Ultimately, the framework's automation and scalability offer a pathway toward personalized, simulation-informed surgical planning for tongue reconstruction.

3. The "Introduction" section should be more concise and some sentences should be rewritten.

Response:
We have extensively revised the Introduction section to improve readability, flow, and conciseness, while ensuring that all relevant background information and citations remain intact. Key changes include streamlined narrative, reduced redundancy and focused literature review. We believe these edits have significantly improved the pacing and clarity of the manuscript's introduction. Thank you for the opportunity!

4. The manuscript is well written, but the Introduction is very concise, and more articles and works need to be discussed and explained. Some other related and interesting topics need to be cited as well, as below:
- "Effect of thermal dispersion on free convection in a fluid saturated porous medium," International Journal of Heat and Fluid Flow, 30, 229-236 (2009). http://dx.doi.org/10.1016/j.ijheatfluidflow.2009.01.004
-Analytical estimation of temperature in living tissues using the TPL bioheat model with experimental verification. Mathematics, 8(7) (2020) http://dx.doi.org/10.3390/math8071188.
-Thermal response of cylindrical tissue induced by laser irradiation with experimental study. International Journal of Numerical Methods for Heat and Fluid Flow, 22(7), 4013-4023 (2020) http://dx.doi.org/10.1108/HFF-10-2019-0777.
-Analytical solutions of thermal damage in living tissues due to laser irradiation. Waves in Random and Complex Media, 31(6), 1443-1456 (2021) http://dx.doi.org/10.1080/17455030.2019.1676934.
-An analytical solution of the bioheat model in a spherical tissue due to laser irradiation. Indian Journal of Physics, 94(9), 1329-1334 (2020) http://dx.doi.org/10.1007/s12648-019-01581-w.
- Effects of magnetohydrodynamic flow past a vertical plate with variable surface temperature. Applied Mathematics and Mechanics (English Edition), 31(3), 329-338 (2010) http://dx.doi.org/10.1007/s10483-010-0306-9

Response:
We sincerely thank the reviewer for highlighting these important related works. We agree that situating our study within the broader landscape of computational tissue modeling strengthens the manuscript significantly. We have carefully reviewed the recommended literature and

expanded our Introduction to incorporate four of the most relevant papers. These citations have been integrated into our introduction on projected tissue behavior, serving to illustrate how analogous computational frameworks (much like ours) are being successfully deployed to model complex physiological phenomena, such as bioheat transfer and laser-induced thermal damage. We believe this addition effectively broadens the scope of our literature review and demonstrates the versatility of computational methods in predicting tissue response to clinical interventions.

Revision:
"... In a broader context, analogous computational frameworks have been established to model bioheat transfer and laser-induced thermal damage in living tissues [33–36]. "

5. The paper is well written and serves the purpose. Except some minor typographical errors there are no major issues.
6. The conclusion must be improve and some sentences should be rewritten.

Response:
We appreciate the positive assessment. We have revised the Conclusion to enhance clarity, flow, and scientific impact as requested. Key sentences were rewritten to use stronger, active voice, and we sharpened the scientific justification by framing our findings as a "quantitative mechanism" for the clinical trade-off between volume and mobility. These edits streamline the reading experience and more effectively underscore the study's implications regarding the coupled effects of donor site selection and overbulking. Thank you for the opportunity.
